# Recent Advances in the Clinical Application of Adrenal Vein Sampling

**DOI:** 10.3389/fendo.2022.797021

**Published:** 2022-02-09

**Authors:** Shan Zhong, Tianyue Zhang, Minzhi He, Hanxiao Yu, Zhenjie Liu, Zhongyi Li, Xiaoxiao Song, Xiaohong Xu

**Affiliations:** ^1^Department of Endocrine and Metabolic Diseases, The Second Affiliated Hospital, Zhejiang University School of Medicine, Hangzhou, China; ^2^Department of Vascular Surgery, The Second Affiliated Hospital School of Medicine, Zhejiang University School of Medicine, Hangzhou, China; ^3^Clinical Research Center, The Second Affiliated Hospital, Zhejiang University School of Medicine, Hangzhou, China; ^4^Department of Urinary Surgery, The Second Affiliated Hospital School of Medicine, Zhejiang University School of Medicine, Hangzhou, China

**Keywords:** adrenal vein sampling (AVS), primary aldosteronism (PA), Cushing syndrome (CS), hyperandrogenism (HA), adrenal

## Abstract

We reviewed clinical research investigating the applications of adrenal vein sampling (AVS). AVS could be applied not only to primary aldosteronism (PA) but also to other endocrine diseases, such as adrenocorticotropic hormone (ACTH) independent Cushing syndrome (AICS) and hyperandrogenemia (HA). However, the AVS protocol requires improvements to increase its success rate. Using the computed tomography image fusion, coaxial guidewire technique, and fast intraprocedural cortisol testing (CCF) technique could improve the success rate of catheterization in AVS for PA. ACTH loading could be considered in medical centers with a low selectivity of AVS for PA but is not essential in those with mature AVS technology. The continuous infusion method should be recommended for ACTH stimulation in AVS for PA to reduce adverse events. AVS has not been routinely recommended before management decisions in AICS, but several studies verified that AVS was useful in finding out the source of excess cortisol, especially for distinguishing unilateral from bilateral disease. However, it is necessary to reassess the results of AVS in AICS with the use of reference hormones to fully normalize cortisol levels. In addition, it is essential to determine the optimal model that combines AVS results and mass size to guide the selection of surgical plans, including identifying the dominant gland and presenting the option of staged adrenalectomy, to minimize the impact of bilateral resection. For HA, AVS combined with ovarian intravenous sampling to locate excess androgens could be considered when imaging results are equivocal.

## Introduction

Adrenal vein sampling (AVS) was first developed in the 1960s (1). The most important and extensive use of AVS was its use as the gold standard method for localizing the overproduction of aldosterone in primary aldosteronism (PA) (2). PA is the most common form of secondary hypertension. The sensitivity and specificity of AVS for detecting unilateral PA (UPA) were 95% and 100%, respectively (2, 3). Moreover, AVS could also be used for other endocrine disorders that are caused by excess secretion of cortisol or androgens from the adrenal gland. Adrenocorticotropic hormone (ACTH) independent Cushing syndrome (AICS) is mostly caused by cortisol-producing adrenocortical tumors. However, in some cases, it is difficult to accurately localize the responsible focuses, especially in the presence of bilateral adrenal masses. AVS might be ideal for distinguishing between the responsible focuses that are causing excess secretion of cortisol. Although hyperandrogenism (HA) is a common endocrine disorder among women, at times, imaging fails to detect the source of overproduction of androgens. Consequently, AVS could be an attractive alternative option for the etiological diagnosis of HA. Theoretically, AVS could be used for pheochromocytoma, although to the best of our knowledge, there are currently no articles on this. Despite AVS being safe and effective, the success rate has varied, ranging from 8 to 95%, which has largely limited its wide application (4–6). Therefore, increasing the use of AVS requires improving the AVS protocol. Furthermore, the reference ranges are still controversial. In addition, the application of AVS in diseases other than PA is not yet known. Hence, in this review, we discussed in detail the latest improvements of AVS use in PA and briefly summarized the expanded application of AVS in AICS and HA, in order to help clinicians better understand the use of AVS in clinical practice. We searched all eligible original articles on PubMed with following keywords: (adrenal vein sampling [Title/Abstract]) AND (primary hyperaldosteronism [Title/Abstract]); (adrenal vein sampling [Title/Abstract]) AND Cushing syndrome [Title/Abstract]); (adrenal vein sampling [Title/Abstract]) AND hyperandrogenism [Title/Abstract]). There were no exclusion criteria in our review because the number of eligible studies was relatively small. The main results of representative studies are presented in **Table 1**.

**Table 1:** Summary of main studies involving advances about application of AVS.

Authors, year	Population	Results	Conclusions	Limitations
Primary Aldosteronism (PA)				
Correct right adrenal vein catheterization				
Liu (7)	105 Patients with PA (51 in the AVS–CCF group; 54 in the AVS group)	The technical success rate was higher for AVS–CCF than for AVS without CCF (98 vs. 83.3% for bilateral adrenal veins, P = 0.016).	The CCF technique during AVS not only contributed to improved technical success rates but also associated with decreased procedure time, radiation exposure, and contrast medium volume.	The AVS–CCF procedures were performed more recently than the AVS without CCF procedures.
Maruyama (8)	90 Patients with PA (43 in the 120-kVp group; 47 in the 70-kVp group)	In comparison with the 120-kVp group, the 70-kVp group had significantly superior conspicuity scores for the RAV (P < 0.001), higher RAV detection rates (P = 0.015–P = 0.033), and lower size-specific dose estimates (P < 0.001).	70-kVp contrast-enhanced CT has advantages over conventional 120-kVp contrast-enhanced CT.	The single-center, retrospective design, and use of 2 different CT scanners and different reconstruction techniques.
The role of ACTH loading during AVS				
Laurent (9)	14 Studies comparing the 2 techniques (AVS with ACTH stimulation and AVS without ACTH stimulation in patient with PA)	AVS with ACTH stimulation significantly reduced the number of unsuccessful cannulations of both adrenal veins more than AVS without ACTH stimulation in patients with PA (OR: 0.26, 95% CI: 0.17, 0.40; P<0.00001).	AVS with ACTH stimulation can significantly reduce the number of unsuccessful cannulations, without significantly reducing the number of incorrect lateralization.	Variability in institutional protocols and shortage of expert interventional radiologists.
Takeda (10)	2197 Japanese patients with PA from 28 centers	ACTH loading during AVS improved the success rate from 67 to 89%, while lateralization indices decreased from 62 to 28%.	The use of ACTH during AVS was helpful for improving the success rate, but did not contribute to better outcomes.	The limitation of the retrospective study.
Hu (11)	174 Patients with PA (80 receiving ACTH bolus; 94 receiving ACTH infusions)	The LI and rate of complete biochemical remission (43/44, 97.7% vs 53/53, 100%, P=0.45) did not significantly differ between the two groups. The bolus group reported more transient AEs such as palpitation (52.9% vs 2.2%) and abdominal discomfort (40.0% vs 2.2%) than the infusion group.	Due to the similar effects on cannulation success and lateralization, but a lower rate of transient AEs in the infusion group, the continuous infusion method should be recommended for ACTH stimulation in AVS.	The adrenal and peripheral venous blood before ACTH administration were not collected.
The evaluation index of AVS results				
Li (12)	37 Patients with PA	SI ≥ 3 for androstenedione or DHEA provided optimal sensitivity and specificity in AVS. Given the much larger AV/PV ratios and reduced variability compared to cortisol, the adrenal androgens are useful for assessing the selectivity of AVS without cosyntropin stimulation.	The adrenal androgens may be superior analytes in conditions with marked variability of cortisol levels or with adrenocortical tumors consecrating cortisol and aldosterone.	The sample size was small and the study did not compare the lateralization indices.
Dekkers (13)	86 Patients with PA (52 in the cosyntropin-stimulated group; 34 in the nonstimulated AVS group)	The adrenal to peripheral vein ratio of metanephrine was 6-fold higher than that of cortisol (94.0 versus 15.5; P<0.0001). ROC analysis indicated a plasma metanephrine SI cutoff of 12.	Metanephrine provides a superior analyte compared with cortisol in assessing the selectivity of adrenal vein sampling during procedures without cosyntropin stimulation.	The sample size was small and the study did not compare the lateralization indices.
Wolley (14)	80 Patients with PA	The degree of contralateral suppression was independently and significantly correlated with postoperative SBP.	Contralateral suppression should be a factor in deciding whether to offer surgery for treatment of PA.	Patients without contralateral suppression were a relatively selected group.
Adrenocorticotropic hormone independent Cushing’s syndrome (AICS)				
Chen (15)	a Case of woman with ACTH-independent ectopic CS	Adrenal CT scan indicated no abnormality. A mass was discovered by pelvic ultrasonography. Combined ovarian and adrenal venous sampling together with a cortisol assay were conducted. Results revealed a right-side ovarian origin.	Combined ovarian and adrenal venous sampling is valuable in the localization of ACTH-independent ectopic CS.	The sample size.
Maghrabi (16)	a Patient with subclinical CS and AIMAH	AVS results were consistent with bilateral autonomous cortisol hypersecretion without lateralization. A left adrenalectomy was performed. The patient improved clinically after the surgery.	AVS is a useful diagnostic tool that helps localize the source of autonomous cortisol hypersecretion in ACTH-independent subclinical CS with bilateral adrenal masses.	The sample size.
Gu (17)	a Patient with CS and BAAs	AVS results were consistent with bilateral autonomous cortisol hypersecretion without lateralization. A left adrenalectomy was performed, followed by resection of the right-side adrenal mass.	AVS is of great significance for obtaining information on the functional state of BAAs before surgery.	The sample size.
Raje (18)	6 Patients with CS (3 with bilateral adrenal enlargement or nodules; 3 with unilateral nodules)	AVS results aided management planning in five patients, definitively changing treatment from surgery to medical management in one patient.	AVS offered useful information for determining appropriate management of adrenal CS.	The sample size.
Hyperandrogenism (HA)				
Tng (19)	3 Studies including women with HA who underwent catherization	The summary sensitivity of the dexamethasone suppression test is 100% and that for selective venous sampling is 100%. The summary specificity of the dexamethasone suppression test is 89.2% and that for selective venous sampling is 100%.	There is limited evidence for the use of selective venous sampling in identifying virilizing tumors in postmenopausal hyperandrogenism.	Poor methodological quality.
Kaltsas (20)	38 Patients who underwent ovarian and adrenal venous catheterization and sampling for investigation of HA	The overall catheterization success rate was: all four veins in 27%, three veins in 65%, two veins in 87%. The success rate for each individual vein was: right adrenal vein 50%, right ovarian vein 42%, left adrenal vein 87% and left ovarian vein 73%.	Venous catheterization and sampling should be considered only for patients in whom uncertainty remains.	The low successful catherization rate.

AVS, adrenal vein sampling; PA, primary aldosteronism; CCF, computed tomography image fusion, coaxial guidewire technique, fast intraprocedural cortisol testing technique; RAV, right adrenal vein; CT, computerized tomography; ACTH, adrenocorticotropic hormone; AE, adverse event; LI, lateralization index; CI, confidence interval; SI, selective index; ROC, receiver operator characteristic; DHEA, dehydroepiandrosterone; AV, adrenal vein; PV, peripheral vein; SBP, systolic blood pressure; CS, Cushing syndrome; AIMAH, ACTH-independent macronodular adrenal hyperplasia; BAAs, bilateral adrenocortical adenomas; HA, hyperandrogenism.

## Primary Aldosteronism

Primary aldosteronism (PA) is the most common cause of secondary hypertension. The prevalence of PA ranges from 3.2 to 12.7% in primary practice and from 1 to 30% in referral centers. This mainly depends on the degree of hypertension in the population being examined. Excess aldosterone is a strong risk factor for heart and kidney damage, independent of sex, age, and blood pressure. The prevalence of PA among patients with recently detected hypertension in China was at least 4%. It is critical to discriminate between the main subtypes to determine the correct therapeutic strategies, which is surgery for unilateral forms or medical therapy for bilateral forms (21–23). There has always been a degree of clinical difficulty in classifying the PA subtypes. Imaging often fails to visualize microadenomas and distinguish nonfunctioning incidentalomas from aldosterone-producing adenomas. The sensitivity and specificity of imaging were 78 and 75%, respectively (2). Clinicians needed to comprehensively analyze the biochemical indicators, imaging manifestations, and AVS results. Despite AVS being the gold standard method and having high sensitivity and specificity for the diagnosis of different primary aldosteronism subtypes, it has varied success rates. Therefore, it is paramount to improve the success rate of AVS.

In 2016, the Endocrine Society Clinical Practice Guideline recommended that for PA, adrenal computed tomography (CT) should be performed before AVS, by an immobilized experienced AVS angiographer, with sufficient time for the operation and with simultaneous bilateral adrenal vein cannulations, while limiting the use of contrast during the procedure to help minimize the failure risk and postoperative complications (2). Many studies focused on increasing the success rate of AVS, including successful right adrenal vein catheterization, the role of ACTH loading during AVS, and the evaluation index of the AVS results, which we have described below.

### Correct Right Adrenal Vein Catheterization

It is well known that AVS failure is often owing to unsuccessful catheterization of the right adrenal vein (the right adrenal vein is usually shorter than the left and typically enters the inferior vena cava (IVC) at an acute angle). A recent study at our center found that the Computed tomography image fusion, Coaxial guidewire technique, Fast intraprocedural cortisol testing (CCF) technique significantly improved technical success rates and reduced procedure time, radiation exposure, and contrast medium volume (7). Previously, the only access for AVS was *via* the femoral vein, but according to the study of J Xu et al., the forearm vein may provide a complementary or alternative approach to catheterization. The success rate of AVS of the right and left adrenal vein was 93.8 and 100%, respectively (24). However, since only 48 patients were included in the study, the reliability of AVS access *via* the forearm vein remained unclear. Another viable method was using imaging to obtain an accurate visualization of the right adrenal vein, which was vital both before and during AVS. With the development of imaging technology, 3-dimensional reconstruction has frequently been applied in clinical settings. Using multi-detector CT (MDCT) with 3-dimensional reconstruction may reduce operation time and the quantity of contrast required and improve the success rate of catheterization. Previous studies have suggested that using Dyna CT, cone-beam CT during AVS could improve successful cannulation of the adrenal vein (25–27). Another study suggested that a 70-kilovoltage-peak (kVp) contrast-enhanced CT scan may provide better visualization and identification of the right adrenal vein (8). Thus, promoting the use of advanced CT techniques at medical centers may be necessary for visualizing the adrenal vein for accurate and successful cannulation.

### The Role of ACTH Loading During AVS

At most medical centers, the medical staff did not possess the skills for simultaneous cannulation and as such, at about 40% of them the use of ACTH infusion during AVS is advocated for to overcome the limitations of non-synchronous catheterization (28, 29). A meta-analysis in 2018 showed that ACTH loading could significantly reduce the number of unsuccessful cannulations, without significantly increasing incorrect lateralization (9). However, a large retrospective study conducted by Tekada et al., in 2019 compared the two techniques (AVS with ACTH stimulation and AVS without ACTH stimulation among 2197 patients with PA from 28 centers in Japan and found that the use of ACTH loading during AVS increased the success rate from 67 to 89%, while decreasing the lateralization rate from 62 to 28% (10). Consequently, ACTH loading could be considered in medical centers with a low selectivity of AVS, but it is not essential in those with mature AVS technology. In addition, a recent study found that the continuous infusion method should be recommended for ACTH stimulation in AVS, due to the similar effects on cannulation success and lateralization, but a lower rate of transient adverse events among patients in the infusion group (11).

### The Evaluation Index of AVS Results

The evaluation of AVS results comprised three indicators, including the selectivity index (SI), lateralization index (LI), and contralateral suppression index (CSI). In 2020, the European Society of Hypertension recommended that the SI of>2 for unstimulated and>5 for stimulated procedures be used to demonstrate correct cannulation of the adrenal veins. The LI of>4 for both unstimulated and stimulated procedures was considered unilateral PA. The CSI of<1 may indicate unilateral PA on the opposite side (22). However, criteria for AVS interpretation may vary between centers, owing to the large heterogeneity in AVS procedures and hormone measurements. Notably, although cortisol-based SI is currently a widely used indicator to evaluate the success of AVS, the secretion of cortisol varies (28, 30, 31). Hence cortisol-based SI may not be the best indicator. With the higher adrenal vein (AV)/peripheral vein (PV) ratio of adrenal androgens, and more importantly, with the lower variability of adrenal androgens than cortisol, adrenal androgen-based SI may be more useful than cortisol-based SI for assessing the selectivity of non-ACTH stimulated AVS. It has also been demonstrated that adrenal androgen-based SI may be superior to cortisol-based SI in adrenal masses producing both aldosterone and cortisol (12). The study also found that the androgen-based SI of≥3 was an optimal cut-off point for assessing the selectivity of AVS. In comparison to catecholamines, there is relatively little increase in metanephrines in response to stress. Furthermore, compared with cortisol, metanephrines provided a superior analyte in assessing the selectivity of AVS, with a SI cut-off point of 12 (13). Although the LI was widely used to determine whether there was dominant secretion of aldosterone, CSI may be a good substitution for some cases where catheterization on the dominant side has been unsuccessful. A retrospective study conducted by Wolley et al., in 2015 showed that the CSI of<1 correlated with positive blood pressure and biochemical outcomes following surgery (14).

## Adrenocorticotropic Hormone Independent Cushing Syndrome

Endogenous Cushing syndrome (CS) is a rare and severe disease with an annual incidence of 0.2 to 5.0 per million and a prevalence of 39 to 79 per million (32). CS has a high mortality rate, with a standard mortality rate (SMR) of approximately 2.0 to 4.0; cardiovascular disease is the most common cause of CS-related death (32). Adrenocorticotropic hormone independent CS (AICS) accounted for about 20% of CS cases, including unilateral adrenal adenoma or carcinoma, bilateral macronodular adrenal hyperplasia, bilateral micronodular adrenal hyperplasia, primary pigmented nodular adrenocortical disease, McCune-Albright syndrome, and bilateral adrenal adenomas or carcinomas. Among patients with AICS with bilateral adrenal masses, it is often difficult to determine the source of excess cortisol, which largely affected surgical planning. However, the source of excess cortisol in AICS could be localized using AVS. Multiple case reports have suggested that AVS may be used to successfully localize excess cortisol in patients with AICS (16, 33–37). In the case of a 40-year-old woman from China with a large pelvic mass, AVS also aided localization of ectopic AICS (15).

The interpretation of AVS results in AICS was controversial. Young et al., stated that epinephrine concentrations exceeding that of PV concentrations by more than 100 pg/mL indicates successful catheterization, an AV/PV cortisol gradient of>6.5 indicates cortisol-secreting adrenal adenoma, and a high-side to low-side AV cortisol lateralization ratio of≥2.3 is consistent with autonomous cortisol secretion from predominantly one adrenal gland (38). A case report showed that a patient with subclinical CS and ACTH-independent macronodular adrenal hyperplasia (AIMAH) underwent AVS and the result indicated no lateralization using criteria stated by Young et al. A left adrenalectomy was performed, and the patient improved clinically after the surgery (the left mass was larger than the right mass) (16). However, in another case with bilateral adrenocortical adenomas (BAAs) and bilateral autonomous cortisol secretion without laterality, the patient required left adrenalectomy followed by the resection of the right-side adrenal mass to gain clinical recovery (17). In both cases with similar AVS results, distinct management was necessary to cure CS, which posed challenges in the interpretation of AVS results, particularly in the question of how to choose the reference hormone to calculate the cortisol to reference hormone ratios from the right and left adrenal veins and finally calculate the lateralization index in non-synchronous catheterization approaches, due to the rapid fluctuation in adrenal hormones during the AVS procedure. In 2018, Wei Jie el at., reported that aldosterone concentrations could be used as reference hormones to calculate the lateralization index (36). However, many factors can interfere with aldosterone concentrations. In addition, aldosterone has a shorter half-life (20 min) than cortisol (60–70 min) that may interfere with the interpretation of AVS findings. Therefore, aldosterone as a reference hormone still requires further validation (33). New criteria stated that a ratio of >12 for metanephrine was considered as correct cannulation. The LI of≥2 was interpreted as unilateral disease, using aldosterone, adrenaline, noradrenaline, and dehydroepiandrosterone sulfate as references (18, 39). Because universal criteria for the evaluation of laterality among patients with hypercortisolism in multi-centers have not yet been established, more multi-center studies are required. Moreover, in clinical practice in the presence of AIMAH, the excision of the larger mass is suggested in order to avoid bilateral adrenalectomy. Thus, it is essential to determine the optimal model that combines AVS results and mass size to guide the selection of surgical plans, to minimize the impact of bilateral resection.

## Hyperandrogenism

Hyperandrogenism (HA) is a common endocrine disorder among women of a reproductive age, with a prevalence of approximately 5-10% (40). Causes of HA included endogenous neoplasms, non-neoplastic androgen overproduction, and exogenous pharmacologic agents. Endogenous neoplasms included adrenocortical adenomas or carcinomas, Sertoli-Leydig cell tumors, hilus cell tumors, teratomas, pituitary adenomas, and others. Adrenal and ovary vein sampling could be considered to locate the source of excess androgen. However, there were only limited reports from studies with small sample size (19, 41–51). A study involving 38 patients with HA showed that the successful catheterization rate of the four veins (bilateral AVs and ovary veins (OVs)) was 27%, and the failure rate of OV catheterization was higher than AV catheterization. OV/PV ratio of estradiol of >2 was considered as successful cannulation of the OV; AV/PV ratio of cortisol of>2 was considered as successful cannulation of the AV; A ratio of testosterone >2 was considered as androgen overproduction (20). In this study, no complications were observed. However, the low success rate of catheterization limited the application of venous sampling in HA, thus it may be only considered when imaging results are equivocal.

## Conclusions

**Figure 1:**
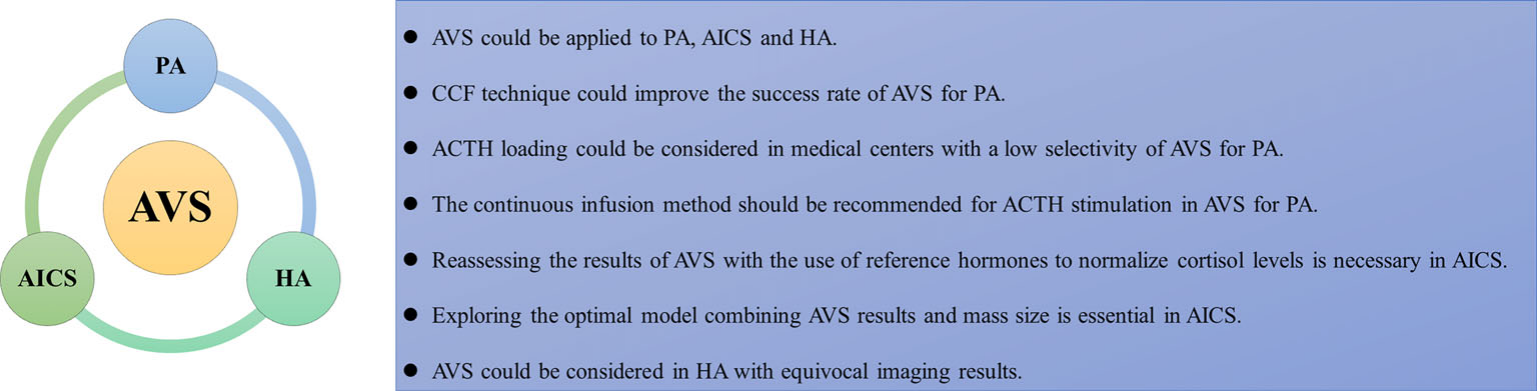
The summarizing figure of the review. AVS, adrenal vein sampling; AICS, adrenocorticotropic hormone independent Cushing syndrome; HA, hyperandrogenism; CCF, computed tomography image fusion, coaxial guidewire technique, fast intraprocedural cortisol testing technique; ACTH, adrenocorticotropic hormone.

Conclusions from our review are presented in a summary figure (**Figure 1**). AVS could be applied not only to PA but also to other endocrine diseases, such as AICS and HA. However, the AVS protocol requires improvements to increase its success rate. Using the CCF technique could improve the success rate of catheterization in AVS for PA. ACTH loading could be considered in medical centers with a low selectivity of AVS for PA but is not essential in those with mature AVS technology. The continuous infusion method should be recommended for ACTH stimulation in AVS for PA to reduce adverse events. AVS hasn’t been routinely recommended before management decisions in AICS, but several studies verified that AVS was useful to find out the source of excess cortisol, especially for distinguishing unilateral from bilateral disease. However, it is necessary to reassess the results of AVS in AICS with the use of reference hormones to fully normalize cortisol levels. In addition, it is essential to determine the optimal model that combines AVS results and mass size to guide the selection of surgical plans, including identifying the dominant gland and presenting the option of staged adrenalectomy to minimize the impact of bilateral resection. For HA, AVS combined with ovarian intravenous sampling to locate excess androgens could be considered when imaging results are equivocal. This study had some limitations. First, since this review only explored the PubMed database and only included articles written in English, some articles may have been missed. Second, most studies included had limited sample sizes. Hence, more research should be conducted to improve the understanding of the clinical application of AVS in endocrine diseases.

## Author Contributions

XS and XX came up with the idea. SZ and TZ wrote the main text. MH, HY, ZheL, and ZhoL collected information. All authors were involved in drafting the manuscript, and have read and approved the final version.

## Funding

This work was supported by the National Natural Science Foundation of China (813000083), the Medicine and Health project founded by Zhejiang Province (2020380946, 2022502078), and the Science and Technology of Zhejiang Province Project (LGF21H070003).

## Conflict of Interest

The authors declare that the research was conducted in the absence of any commercial or financial relationships that could be construed as a potential conflict of interest.

## Publisher’s Note

All claims expressed in this article are solely those of the authors and do not necessarily represent those of their affiliated organizations, or those of the publisher, the editors and the reviewers. Any product that may be evaluated in this article, or claim that may be made by its manufacturer, is not guaranteed or endorsed by the publisher.
